# Prognostic analysis of radiation pneumonitis: carbon-ion radiotherapy in patients with locally advanced lung cancer

**DOI:** 10.1186/s13014-017-0830-z

**Published:** 2017-05-30

**Authors:** Kazuhiko Hayashi, Naoyoshi Yamamoto, Masataka Karube, Mio Nakajima, Naruhiro Matsufuji, Hiroshi Tsuji, Kazuhiko Ogawa, Tadashi Kamada

**Affiliations:** 1 Hospital of the National Institute of Radiological Sciences, National Institutes for Quantum and Radiological Sciences and Technology, 4-9-1 Anagawa, Inage Ward, Chiba City, Chiba Prefecture Japan; 2 Department of Radiotherapy, Mitsui Memorial Hospital, Tokyo, Japan; 3 Department of Accelerator and Medical Physics, Research Center for Particle Therapy, National Institute of Radiological Sciences, Chiba, Japan; 4 Department of Radiation Oncology, Osaka University Graduate School of Medicine, Osaka, Japan

**Keywords:** Radiation pneumonitis, Carbon-ion radiotherapy, Lung cancer

## Abstract

**Background:**

Carbon-ion radiotherapy (CIRT) is a promising treatment for locally advanced non-small-cell lung cancer, especially for patients with inoperable lung cancer. Although the incidence of CIRT-induced radiation pneumonitis (RP) ≥ grade 2 ranges from 2.5 to 9.9%, the association between CIRT-induced RP and dosimetric parameters is not clear. Herein, we identified prognostic factors associated with symptomatic RP after CIRT for patients with non-small-cell lung cancer.

**Methods:**

Clinical results of 65 patients treated with CIRT between 2000 and 2015 at the National Institute of Radiological Sciences were retrospectively analyzed. Clinical stage II B disease (TNM classification) was the most common stage among the patients (45%). The median radiation dose was 72 Gy (68–76) relative biological effectiveness (RBE) in 16 fractions. In cases involving metastatic lymph nodes, prophylactic irradiation of mediastinal lymph nodes was performed at a median dose of 49.5 Gy (RBE). The median follow-up was 22 months.

**Results:**

Grade 2 and grade 3 RP occurred in 6 and 3 patients (9 and 5%), respectively. No patients developed grade 4 or 5 RP. Using univariate analysis, vital capacity as a percentage of predicted (%VC), forced expiratory volume in 1 s (FEV1), mean lung dose (MLD), volume of lung receiving ≥5 Gy (RBE) (V_5_), V_10_, V_20_ and V_30_ were determined to be the significant predictive factors for ≥ grade 2 RP. The receiver operating characteristic (ROC) analysis revealed the cutoff values for %VC, FEV1, MLD, V_5_, V_10_, V_20_ and V_30_ for ≥ grade 2 RP, which were 86.9%, 1.16 L, 12.5 Gy (RBE), 28.8, 29.9, 20.1 and 15.0%, respectively. In addition, the multivariate analysis revealed that %VC <86.9% (odds ratio = 13.7; *p* = 0.0041) and V_30_ ≥ 15% (odds ratio = 6.1; *p* = 0.0221) were significant risk factors.

**Conclusions:**

Our study demonstrated the risk factors for ≥ grade 2 RP after carbon-ion radiotherapy for patients with locally advanced lung cancer.

## Introduction

Primary lung cancer is one of the most common cancers worldwide, and the most frequent type is non-small-cell lung cancer. Patients with locally advanced non-small-cell lung cancer undergo surgery, chemotherapy, and/or radiotherapy. For elderly patients or patients with serious comorbidities, radiotherapy is often the chosen treatment [1, 2]. Carbon-ion radiotherapy (CIRT) is one type of radiotherapy, and its use is spreading throughout Europe and Asia. Compared with photon radiotherapy, CIRT has the following advantages: 1) high doses can be prescribed for tumors with avoidance of the surrounding normal tissue because the dose can be locally concentrated; and 2) the relative biological effectiveness (RBE) is high, with particular efficacy for hypoxic or photon-resistant tumors.

Radiation pneumonitis (RP) is a radiation-induced pulmonary injury. RP generally occurs between 1 month and 1 year after radiotherapy. For photon radiotherapy in lung cancer patients, the incidence of symptomatic RP has ranged from 17 to 37% [3–7], and the incidence of CIRT-induced RP ≥ grade 2 has ranged from 2.5 to 9.9% [8, 9]. Symptomatic RP patients sometimes require treatment with steroids or oxygenation (that is, in patients with ≥ grade 2 RP), and RP is occasionally life-threatening. Thus, it is necessary to reduce the incidence of severe RP.

As for predictors associated with ≥ grade 2 RP, many studies regarding photon radiotherapy have already reported that RP risk depends on irradiated lung volume and dose. The predictive dosimetric parameters are mean lung dose (MLD), V_13_, V_20_, V_30_ and others [3–5, 10]. In addition, other clinical factors such as performance status, age, chemotherapy, tumor site and smoking history have been shown as predictive factors [11–13]. By contrast, only one study concerning CIRT for stage I lung cancer reported predictive factors for ≥ grade 2 RP as follows: respiratory-gate irradiation, irradiation portals with opposing fields, and the maximum dose employed [14]. As a result, the association between dosimetric parameters and RP is not clear in terms of patients with locally advanced lung cancer who have been treated with CIRT. Thus, we analyzed the association between ≥ grade 2 RP and risk factors in detail, identifying the prognostic factors using the data from patients with locally advanced non-small-cell lung cancer who underwent carbon-ion radiotherapy.

## Patients and methods

### Patients

This study was performed in accordance with the guidelines approved by the institutional review board of our institution. This study was a retrospective evaluation of all 141 patients who were treated with CIRT at the National Institute of Radiological Sciences (NIRS). Of these patients, 124 individuals who received CIRT between April 2000 and July 2015 were selected for the study. Eligibility criteria were as follows: 1) patients were diagnosed with non-small-cell carcinoma lung cancer by histology or cytology; 2) the clinical stage had been decided by imaging (computed tomography (CT), magnetic resonance imaging (MRI) or positron emission tomography (PET)); 3) the clinical stage ranged from II A to III B according to the Unio Internationalis Contra Cancrum TNM Classification 7^th^ edition, and the N stage was N0, N1 or N2 [15]; 4) the performance status was 0–2; 5) patients were not treated with chemotherapy within 4 weeks of initiation of CIRT; 6) patients were not suffering from other active cancers; 7) the estimated life expectancy was longer than 6 months; 8) patients had not received previous thoracic radiotherapy; 9) patients were not able to receive other curative therapy (surgery, chemotherapy, and/or radiotherapy) or refused it; 10) patients’ CT and planning data were available for this analysis; and 11) patients received follow-up for more than 6 months at the time of the analysis. This study included 92 patients who met our criteria. Of these patients, 27 of them were excluded because they underwent a second CIRT treatment for recurrent or newly developed lesions in the lung, mediastinal lymph nodes or bone. In addition, 5 patients were excluded because their follow-up periods were less than 6 months. Of these five patients, four patients died of causes unrelated to the treatment, and one patient did not attend the follow-up appointments. Consequently, we retrospectively analyzed the clinical results and treatment plans of 65 patients with locally advanced non-small-cell lung cancer.

### Carbon-ion radiotherapy

The CT images for all patients, which were fixed by an individually tailored immobilization device (Moldcare; Alcare, Tokyo, Japan; and Shelfitter; Kuraray, Osaka, Japan), were taken in the supine or the prone position, with the respiratory system. The CT images were used to develop a 3-dimensional treatment plan using in-house HIPLAN software (NIRS, Chiba, Japan) until the end of 2011, and XiO-N (ELEKTA, Stockholm, Sweden and Mitsubishi Electric, Tokyo, Japan) was used beginning in 2012.

Carbon-ion beams were generated by the heavy ion medical accelerator in the Chiba (HIMAC) synchrotron and were delivered using the respiratory-gated irradiation system [16]. Irradiation was performed in 3–4 fields with 250 MeV or 290 MeV carbon ions. The most common prescribed dose was 72 Gy (RBE) in 16 fractions (45 patients, 69%), followed by 76 Gy (RBE) in 16 fractions (11 patients, 17%) and 68 Gy (RBE) in 16 fractions (9 patients, 14%). All doses were administered 4 days per week over 4 weeks.

Primary lesions and metastatic lymph nodes were contoured as gross tumor volume (GTV) on the CT images. The primary lesions with a 10-mm margin and any prophylactic lymph nodes were defined as the clinical target volume (CTV). For N0 cases, irradiation to prophylactic lymph nodes was omitted. Planning target volume (PTV) was defined as the CTV plus a 5-mm safety margin to account for position uncertainty. The dose was prescribed to the isocenter. The PTV was enclosed conformally at a minimum by the 95% isodose line with the prescribed dose. If there were metastatic lymph nodes, prophylactic irradiation to mediastinal lymph nodes was performed at a median dose of 49.5 Gy (RBE). Maximum dose constraints were as follows: main bronchus, 60 Gy (RBE); esophagus, 50 Gy (RBE); and spinal cord, 30 Gy (RBE).

The dose calculation algorithm at our hospital was updated from the broad-beam calculation using in-house HIPLAN software to the pencil-beam calculation using the XiO-N software in 2012. Different dose calculation algorithms cause different dose distributions for the same treatment. For the current study, the treatment plans calculated by the HIPLAN software were converted to DICOM format and imported into XiO-N. Their dose distributions were recalculated with XiO-N.

### Clinical and dosimetric analysis

First, dosimetric parameters (PTV, MLD, V_5_, V_10_, V_20_, V_30_, V_40_ and V_50_) were calculated with XiO-N using the bilateral lung volume-GTV as the all-lung volume. Second, clinical parameters (patient characteristics and pulmonary function) and dosimetric parameters associated with ≥ grade 2 RP were identified using univariate analysis. Third, cutoff values of these parameters were determined using the receiver operating characteristic (ROC) curve. Finally, the predictors of ≥ grade 2 RP were identified using multivariate analysis.

### Evaluation of radiation pneumonitis

The severity of RP was evaluated according to The National Cancer Institute’s Common Terminology Criteria for Adverse Events version 4.0 [17]. For example, we classified the initiation of required steroids as grade 2 and oxygen induction as grade 3.

### Follow-up

After completion of treatment, follow-up observations were performed at 1, 3, 6, 9 and 12 months, and then every 3 or 6 months if serious complications did not occur. At follow-up, CT images, blood examination and respiratory function assessments were performed and, if necessary, MRI brain images and PET/CT were added.

### Statistical analysis

We performed univariate analysis using Fisher’s exact test or Mann–Whitney U test. Multivariate analysis was performed using the Cox proportional hazard model. Statistical significance was set at p < 0.05. The ROC curve and the Youden index were calculated to determine cutoff values. We used JMP statistical software (version 11.0) for all statistical analyses.

## Results

### Patient characteristics

The median follow-up period for all 65 patients was 22.0 months (6.0–145.7 months). The patients consisted of 51 males and 14 females (median age at treatment, 74 years; range, 46–88) (Table 1). Disease sites included 23 right upper lobes, 17 right lower lobes, 16 left upper lobes, 5 left lower lobes and 4 right middle lobes. The clinical stage was II A disease in 8 patients, II B disease in 29 patients, III A in 16 patients, and III B in 12 patients, according to the TNM classification system. Fourteen patients (22%) received chemotherapy before or after CIRT. No patients received CIRT and chemotherapy concurrently. Fifty-six patients (86%) were current or previous smokers.

**Table 1:** Patient characteristics

Factors	Number (%)
Age (years)	
median (range)	74 (46–88)
Sex	
Male	51 (78)
Female	14 (22)
PS	
0	22 (34)
1	39 (60)
2	4 (6)
Location of primary tumor	
Right upper lobe	23 (35)
Right middle lobe	4 (6)
Right lower lobe	17 (26)
Left upper lobe	16 (25)
Left lower lobe	5 (8)
Clinical Stage	
II A	8 (12)
II B	29 (45)
III A	16 (25)
III B	12 (18)
Histology	
Squamous cell carcinoma	35 (54)
Adenocarcinoma	26 (40)
Large cell carcinoma	2 (3)
Non small-cell carcinoma	2 (3)
Total dose	
68 Gy (RBE)/16 fr	9 (14)
72 Gy (RBE)/16 fr	45 (69)
76 Gy (RBE)/16 fr	11 (17)
Chemotherapy	
Yes	14 (22)
No	51 (78)
Smoking status	
Current or previous	56 (86)
Never	9 (14)
Pulmonary emphysema	
Yes	12 (18)
No	53 (82)

*PS* performance status, *RBE* relative biological effectiveness, *fr* fractions

### Incidence of radiation pneumonitis (RP)

Table 2 shows the number of patients according to RP grade. No patients developed ≥ grade 4 RP. Grade 3 RP occurred in 3 patients (5%) at approximately 4–6 months after the initiation of CIRT. These patients were prescribed steroids and home oxygen therapy. The prescribed doses were 72 Gy (RBE) for 2 patients and 76 Gy (RBE) for 1 patient. Grade 2 RP occurred in 6 patients (9%) at a median of 5 months (5–13 months), and all patients were treated with steroids. The prescribed doses were 76 Gy (RBE) for 1 patient, 72 Gy (RBE) for 4 patients, and 68 Gy (RBE) for 1 patient. In total, 9 patients (14%) suffered from ≥ grade 2 RP.

**Table 2:** Number of patients with radiation pneumonitis

	G0	G1	G2	G3	G4 and G5
Number of patients	32	24	6	3	0

*G* Grade

### Clinical factors associated with ≥ grade 2 RP

Univariate analysis results of patient characteristics with or without ≥ grade 2 RP are shown in Table 3. There were no patient characteristic factors associated with ≥ grade 2 RP. The incidence of ≥ grade 2 RP tended to be higher for the ≥75 years of age group than for patients <75 years of age, although the difference was not statistically significant.

**Table 3:** Univariate analysis of patient characteristics

Parameters	No of patients with ≥ G2 RP (%)	No of patients with G0-1 RP (%)	*p* value
Age			0.074
< 75 years old	1 (11)	25 (45)	
≥ 75	8 (89)	31 (55)	
Sex			0.392
Man	6 (67)	45 (80)	
Female	3 (33)	11 (20)	
PS			0.458
0 or 1	8 (89)	53 (95)	
2	1 (11)	3 (5)	
Location of primary tumor			0.706
Upper or Middle lobe	7 (78)	36 (64)	
Lower lobe	2 (22)	20 (36)	
Clinical Stage			1.000
II	5 (56)	32 (57)	
III	4 (44)	24 (43)	
Total dose			1.000
≤ 72 Gy RBE	8 (89)	46 (82)	
> 72 Gy RBE	1 (11)	10 (18)	
Chemotherapy			0.187
Yes	0	14 (25)	
No	9 (100)	42 (75)	
Brinkman Index			1.000
< 600	3 (33)	19 (34)	
≥ 600	6 (67)	37 (66)	
Pulmonary emphysema			0.191
Yes	0	12 (21)	
No	9 (100)	44 (79)	

*G* grade, *RP* radiation pneumonitis, *PS* performance status, *RBE* relative biological effectiveness

Univariate analysis of the average pulmonary function was performed to explore potential prognosticators for ≥ grade 2 RP (Table 4). All data regarding pulmonary function were evaluated as continuous variables. The results showed that low percentage of vital capacity (%VC) and 1-second forced expiratory volume (FEV1) values were significant prognostic factors for ≥ grade 2 RP (%VC: *p* = 0.002; FEV1: *p* = 0.043). The ROC analysis was used to determine the cutoff values for %VC (86.9%) and FEV1 (1.16 L) for ≥ grade 2 RP. Patients were divided into two groups according to the cutoff values, and the actual incidences of ≥ grade 2 RP were 26.5% for %VC <86.9% and 0% for %VC ≥86.9% (*p* = 0.002). Similarly, the cutoff values were 6.8% for FEV1 ≥ 1.16 and 28.6% for FEV1 < 1.16 (*p* = 0.048).

**Table 4:** Univariate analysis of average pulmonary function

Parameters	≥grade 2 (min - max)	grade 0–1 (min - max)	*p* value
%VC (%)	67.5 (37.1–86.9)	89.2 (53.2–120.8)	0.002
FEV1 (L)	1.17 (0.5–1.78)	1.67 (0.6–3.33)	0.043
FEV1/FVC (%)	70.0 (56.2–83.7)	65.6 (34.2–99.7)	0.556
%DLCO (%)	72.6 (32.8–123.9)	78.0 (29.5–178.4)	0.644

*%VC* percent of vital capacity, *FEV1* 1-second forced expiratory volume, *FVC* forced vital capacity, *%DLCO* percent of diffusing capacity for carbon monoxide

### Dose-volume analysis of ≥ grade 2 RP

The dose-volume parameters associated with ≥ grade 2 RP were analyzed (Table 5). The results illustrated that the mean lung dose (MLD), the volume of lung receiving ≥5 Gy (RBE) (V_5_), V_10_, V_20_ and V_30_ were significant predictive factors for ≥ grade 2 RP. The ROC analysis was used to determine the cutoff values for MLD, V_5_, V_10_, V_20_ and V_30_ for ≥ grade 2 RP, which were 12.5 Gy (RBE), 28.8, 29.9, 20.1 and 15.0%, respectively. The actual incidences of ≥ grade 2 RP were 35.7% vs. 7.8% (MLD, ≥12.5 Gy (RBE) vs. less than 12.5 Gy (RBE), respectively, *p* = 0.018), 24.1% vs. 5.6% (V_5_, ≥28.8% vs. less than 28.8%, respectively, *p* = 0.066), 26.1% vs. 7.1% (V_10_, ≥29.9% vs. less than 29.9%, respectively, *p* = 0.058), 21.9% vs. 5.9% (V_20_, ≥20.1% vs. less than 20.1%, *p* = 0.074), and 28.0% vs. 0% (V_30_, ≥15.0% vs. less than 15%, *p* = 0.022).

**Table 5:** Univariate analysis of average dosimetric parameters

Parameters	≥ grade 2 (min - max)	grade 0–1 (min - max)	*p* value
PTV (cm^3^)	448.5 (155.8–712.7)	371.0 (73.8–1319.8)	0.193
MLD (Gy RBE)	12.0 (6.5–17.0)	9.0 (2.9–16.2)	0.023
V_5_ (%)	33.8 (17.9–49.0)	24.9 (7.0–46.1)	0.034
V_10_ (%)	30.6 (15.3–46.2)	22.6 (6.5–42.6)	0.045
V_20_ (%)	24.5 (10.1–31.4)	17.9 (5.6–36.7)	0.039
V_30_ (%)	19.0 (10.1–31.4)	12.9 (3.7–23.6)	0.015
V_40_ (%)	12.2 (5.2–18.1)	8.8 (3.1–18.9)	0.052
V_50_ (%)	6.7 (2.6–13.3)	5.8 (2.1–14.2)	0.442

*PTV* planning target volume, *MLD* mean lung dose, *RBE* relative biological effectiveness, *V*_*X*_ volume of lung receiving ≥ X Gy (RBE)

### Multivariate analysis of risk factors for ≥ grade 2 RP

Multivariate analysis was performed for ≥ grade 2 RP using the two variables with the most significant *p* values from the univariate analysis of the clinical, pulmonary functional or dosimetric factors (Table 6). The results showed that %VC (odds ratio = 13.7; *p* = 0.0041) and V_30_ (odds ratio = 6.1; *p* = 0.0221) were significant prognosticators for ≥ grade 2 RP.

**Table 6:** Multivariate analysis of risk factors for ≥ grade 2 radiation pneumonitis

	≥grade 2 Radiation Pneumonitis	
Factor	Odds Ratio (95% CI)	*p* value
%VC (<86.9% vs ≥86.9%)	13.7 (2.09–276.2)	0.0041
V_30_ (≥15% vs <15%)	6.1 (1.29–36.3)	0.0221

*RP* radiation pneumonitis, *%VC* percent of vital capacity, *RBE* relative biological effectiveness, *V*_*30*_ volume of lung receiving ≥30 Gy RBE

## Discussion

Carbon-ion radiotherapy in patients with locally advanced non-small-cell lung cancer is a promising treatment, especially for patients with inoperable lung cancer [8]. Generally, RP is a serious adverse effect of thoracic CIRT. Takahashi et al. conducted a phase I/II prospective study to investigate the safety and efficacy of CIRT in 62 patients with locally advanced non-small-cell lung cancer and reported an 8.1% incidence rate of ≥ grade 2 RP [8]. Our study was a retrospective study that analyzed the risk factors for ≥ grade 2 RP in 65 selected patients from an initial cohort of 141 patients and showed an incidence of grade 2/3 RP in 9 of 65 patients (14%), and no patients developed grade 4/5 RP. The fact that our incidence rate was slightly higher than that of Takahashi’s et al. may be attributed to a difference in eligibility criteria. Regarding photon radiotherapy in lung cancer patients, many previous studies reported rates of symptomatic RP ranging from 17 to 37% [3–7]; more current research involving photon radiotherapy alone has not reported an incidence rate of symptomatic RP. One recent study, the RTOG 0617 study concerning chemoradiotherapy, showed an 8.3% incidence rate of ≥ grade 2 RP. [18]. The incidence of RP from modern photon radiotherapy tended to decrease because of the accumulation of dosimetric findings and technical advances. Similarly, our results concerning CIRT-induced RP may decrease the incidence of RP in the future. We conducted this study to further improve the safety of CIRT for patients with locally advanced lung cancer.

Barriger et al. reported that symptomatic RP developed at a median of 3.5 months (0.5-12 months) after the initiation of treatment [19]. Our study showed that ≥ grade 2 RP occurred at a median of 5 months after the initiation of CIRT. Our result was comparable to Barriger’s result from photon radiotherapy regarding the onset of RP.

According to the reported studies of photon radiotherapy, several prognosticators such as concurrent chemotherapy, age, MLD, V_13_, V_20_, V_30_ and others were identified [3, 4, 10, 20, 21]. Multivariate analysis of our study revealed that the significant prognostic factors for ≥ grade 2 RP were %VC and V_30_. While V_30_ had already been identified as a prognostic factor for RP in photon therapy, in this study, %VC prior to treatment was identified as an independent prognostic factor for RP. This may arise from the fact that our study included patients with very low pulmonary function, and they tended to advance in RP severity. In fact, the average %VC of patients with ≥ grade 2 RP was 67.5%, and that of patients without ≥ grade 2 RP was 89.2%. When patients with a low %VC suffer from RP, dyspnea often appears or the saturation percentage of oxygen in arterial blood deteriorates easily because of low vital capacity. Consequently, they need to be treated with steroids or oxygenation (that is, in patients with ≥ grade 2 RP). In the future, our findings may suggest that a dose constraint of V_30_ < 15% be imposed on treatment planning, and that when patients with %VC < 86.9% are treated, special care must be provided during follow-up.

Our study had 2 limitations. First, only 9 patients developed ≥ grade 2 RP because CIRT-induced RP is relatively rare [8]. Second, it was difficult to accurately distinguish RP from other types of pneumonitis because RP is a clinical diagnosis and can be confounded by preexisting or comorbid disease, including chronic obstructive pulmonary disease exacerbations, cardiac disease, tumor progression or infection [19, 22].

To further reduce the incidence of ≥ grade 2 RP, our results should influence treatment considerations. For example, for CIRT for lung cancer patients whose %VC is less than 86.9%, a dose constraint of V_30_ less than 15% should be imposed. In the future, when clinical trials of concurrent chemotherapy and CIRT are planned, these results should be informative for treatment decisions.

## Conclusions

Our study identified %VC <86.9% (odds ratio = 13.7) and V_30_ ≥ 15% (odds ratio = 6.1) as significant risk factors for ≥ grade 2 RP. Our study was a single institutional retrospective analysis, and further multi-institutional prospective studies are warranted.

## Abbreviations

%DLCO: Percent of diffusing capacity for carbon monoxide
%VC: Percent of vital capacity
CIRT: Carbon-ion radiotherapy
CT: Computed tomography
CTV: Clinical target volume
FEV1: 1-second forced expiratory volume
fr: Fractions
FVC: Forced vital capacity
G: Grade
GTV: Gross tumor volume
HIMAC: The Heavy Ion Medical Accelerator in Chiba
MLD: Mean lung dose
MRI: Magnetic resonance imaging
NIRS: National Institute of Radiological Sciences
PET: Positron emission tomography
PS: Performance status
PTV: Planning target volume
RBE: Relative biological effectiveness
ROC: Receiver operating characteristic
RP: Radiation pneumonitis
VX: Volume of lung receiving ≥ X Gy (RBE)

## References

[1] Hayakawa K, Mitsuhashi N, Katano S, Saito Y, Nakayama Y, Sakurai H (2001). High-dose radiation therapy for elderly patients with inoperable or unresectable non-small cell lung cancer. Lung Cancer.

[2] Reinfuss M, Glinski B, Kowalska T, Kulpa J, Zawila K, Reinfuss K (1999). Radiotherapy for stage III, inoperable, asymptomatic small cell lung cancer. Final results of a prospective randomized study (240 patients). Cancer Radiother.

[3] Graham MV, Purdy JA, Emami B, Harms W, Bosch W, Lockett MA (1999). Clinical dose–volume histogram analysis for pneumonitis after 3D treatment for non-small cell lung cancer (NSCLC). Int J Radiat Oncol.

[4] Hernando ML, Marks LB, Bentel GC, Zhou SM, Hollis D, Das SK (2001). Radiation-induced pulmonary toxicity: a dose-volume histogram analysis in 201 patients with lung cancer. Int J Radiat Oncol Biol Phys.

[5] Kwa SL, Lebesque JV, Theuws JC, Marks LB, Munley MT, Bentel G (1998). Radiation pneumonitis as a function of mean lung dose: an analysis of pooled data of 540 patients. Int J Radiat Oncol Biol Phys.

[6] Oetzel D, Schraube P, Hensley F, Sroka-Pérez G, Menke M, Flentje M (1995). Estimation of pneumonitis risk in three-dimensional treatment planning using dose-volume histogram analysis. Int J Radiat Oncol Biol Phys.

[7] Sunyach MP, Falchero L, Pommier P, Perol M, Arpin D, Vincent M (2000). Prospective evaluation of early lung toxicity following three-dimensional conformal radiation therapy in non-small-cell lung cancer: preliminary results. Int J Radiat Oncol Biol Phys.

[8] Takahashi W, Nakajima M, Yamamoto N, Yamashita H, Nakagawa K, Miyamoto T (2015). A prospective nonrandomized phase I/II study of carbon ion radiotherapy in a favorable subset of locally advanced non-small cell lung cancer (NSCLC). Cancer.

[9] Miyamoto T, Baba M, Sugane T, Nakajima M, Yashiro T, Kagei K (2007). Carbon ion radiotherapy for stage I non-small cell lung cancer using a regimen of four fractions during 1 week. J Thorac Oncol.

[10] Kong F-M, Hayman JA, Griffith KA, Kalemkerian GP, Arenberg D, Lyons S (2006). Final toxicity results of a radiation-dose escalation study in patients with non-small-cell lung cancer (NSCLC): predictors for radiation pneumonitis and fibrosis. Int J Radiat Oncol Biol Phys.

[11] Quon H, Shepherd FA, Payne DG, Coy P, Murray N, Feld R (1999). The influence of age on the delivery, tolerance, and efficacy of thoracic irradiation in the combined modality treatment of limited stage small cell lung cancer. Int J Radiat Oncol Biol Phys.

[12] Robnett TJ, Machtay M, Vines EF, McKenna MG, Algazy KM, McKenna WG (2000). Factors predicting severe radiation pneumonitis in patients receiving definitive chemoradiation for lung cancer. Int J Radiat Oncol Biol Phys.

[13] Yamada M, Kudoh S, Hirata K, Nakajima T, Yoshikawa J (1998). Risk factors of pneumonitis following chemoradiotherapy for lung cancer. Eur J Cancer.

[14] Miyamoto T, Yamamoto N, Nishimura H, Koto M, Tsujii H, Mizoe J (2003). Carbon ion radiotherapy for stage I non-small cell lung cancer. Radiother Oncol.

[15] Sobin LH, Gospodarowicz MK, Wittekind C (2009). International union against cancer. TNM classification of malignant tumours.

[16] Minohara S, Kanai T, Endo M, Noda K, Kanazawa M (2000). Respiratory gated irradiation system for heavy-ion radiotherapy. Int J Radiat Oncol Biol Phys.

[17] Services UDOHAH. Common Terminology Criteria for Adverse Events v4.0. NIH publication. 2009. https://evs.nci.nih.gov/ftp1/CTCAE/CTCAE_4.03_2010-06-14_QuickReference_5x7.pdf.

[18] Bradley JD, Paulus R, Komaki R, Masters G, Blumenschein G, Schild S (2015). Standard-dose versus high-dose conformal radiotherapy with concurrent and consolidation carboplatin plus paclitaxel with or without cetuximab for patients with stage IIIA or IIIB non-small-cell lung cancer (RTOG 0617): a randomised, two-by-two factorial phase 3 study. Lancet Oncol.

[19] Barriger RB, Forquer JA, Brabham JG, Andolino DL, Shapiro RH, Henderson MA (2012). A dose-volume analysis of radiation pneumonitis in non-small cell lung cancer patients treated with stereotactic body radiation therapy. Int J Radiat Oncol Biol Phys.

[20] Byhardt RW, Scott C, Sause WT, Emami B, Komaki R, Fisher B (1998). Response, toxicity, failure patterns, and survival in five Radiation Therapy Oncology Group (RTOG) trials of sequential and/or concurrent chemotherapy and radiotherapy for locally advanced non-small-cell carcinoma of the lung. Int J Radiat Oncol Biol Phys.

[21] Claude L, Pérol D, Ginestet C, Falchero L, Arpin D, Vincent M (2004). A prospective study on radiation pneumonitis following conformal radiation therapy in non-small-cell lung cancer: clinical and dosimetric factors analysis. Radiother Oncol.

[22] Kocak Z, Evans ES, Zhou S-M, Miller KL, Folz RJ, Shafman TD (2005). Challenges in defining radiation pneumonitis in patients with lung cancer. Int J Radiat Oncol Biol Phys.

